# Early Discontinuation of Empiric Antibiotic Therapy in Children with Cancer and Febrile Neutropenia: A Narrative Review

**DOI:** 10.3390/medicina62010103

**Published:** 2026-01-02

**Authors:** Smaragda Papachristidou, Dimitra Dimopoulou, George Pantalos, Dimitrios Doganis, Sophia Pasparaki, Lydia Kossiva, Vassiliki Papaevangelou, Maria Tsolia

**Affiliations:** 1 Second Department of Paediatrics, School of Medicine, National and Kapodistrian University of Athens (NKUA), P.&A. Kyriakou Children’s Hospital, 11527 Athens, Greece; smagia.gsp@gmail.com (S.P.); spasparaki97@gmail.com (S.P.); lydiakossiva@hotmail.com (L.K.); mariantsolia@gmail.com (M.T.); 2 Department of Paediatric Oncology, P.&A. Kyriakou Children’s Hospital, 11527 Athens, Greece; doganisd@gmail.com; 3 Pediatric Surgery Department, Penteli’s Children Hospital, 15236 Penteli, Greece; gpantalos@gmail.com; 4 Third Department of Paediatrics, School of Medicine, National and Kapodistrian University of Athens (NKUA), Attikon Hospital, 12462 Athens, Greece; vpapaev@gmail.com

**Keywords:** neutropenia febrile, discontinuation early, discharge early, antibiotics, cohort prospective, cancer pediatric

## Abstract

*Background and Objectives*: Febrile neutropenia (FN) is a potentially life-threatening complication in children undergoing cancer treatment. Immediate initiation of empirical antibiotic treatment (EAT) has improved the prognosis and outcomes of FN. Although the ideal timing for initiating EAT is clear, the optimal timing for EAT discontinuation remains debatable. Early hospital discharge (EHD) with continuation of oral antibiotics has also been proposed as an alternative strategy. This narrative review aims to present a comprehensive overview of the evidence on early discontinuation of EAT or EHD in children with FN and cancer. *Materials and Methods*: A comprehensive literature search was performed to identify relevant studies assessing early EAT discontinuation or EHD in children with cancer and FN. Extracted data included the safety outcomes, the benefits for the patients and the cost for healthcare systems. *Results*: Thirty-one studies were included; twenty-one investigated the early discontinuation of EAT and ten studies evaluated EHD. Most studies reported early discontinuation of EAT or EHD as a safe FN treatment approach with potential benefits for the patients, especially when applied to selected low-risk FN cases. Reported benefits included shorter hospitalization duration and reduced antibiotic use, with additional economic advantages in several studies. *Conclusions*: Early discontinuation of EAT appears to be a safe and beneficial management approach for children with FN and cancer by reducing the length of hospital stay and the duration of antibiotic use. EHD with oral therapy continuation also appears to be safe but less beneficial than early discontinuation of EAT. High-quality evidence and standardized criteria are needed to support broader implementation of these strategies in routine clinical practice.

## 1. Introduction

Febrile neutropenia (FN) remains a common and potentially life-threatening complication in children undergoing treatment for cancer. In the United States, there is a reported FN incidence of 7.8 cases per 1000 pediatric oncology patients, [1] while in Europe the reported incidence is 8 FN cases per 1000 patients with cancer [2]. Mortality rate varies from 0.4% to 0.8% in recent studies [3,4,5,6]. Early administration of broad-spectrum empirical antibiotic therapy (EAT) has significantly improved outcomes, especially in cases presenting with bacteremia and/or septicemia [7,8]. The ideal timing for the initiation of EAT is well-established by the definition of the “golden hour”, namely the initiation of EAT within the first 60 min from arrival to the hospital [9].

However, the optimal timing for EAT discontinuation remains debatable. During past years, the continuation of EAT until neutrophil recovery was the standard of care, based on the evidence that higher neutrophil counts result in the adequate immunological response to infection [10]. Consequently, the standard approach was hospitalization and administration of EAT until neutrophil recovery—either absolute neutrophil count (ANC) > 0.5 × 10^3^/μL or at least 7 days of EAT [11,12]. Thus, this approach is associated with prolonged length of hospital stay (LOS), with the potential risk of hospital-acquired infections, extended antibiotic courses, with potential implications for antimicrobial resistance, and increased healthcare cost. Furthermore, delays in chemotherapy administration have been reported in rates as high as 13.3% of children with FN [13]. Prolonged hospitalization appears to be an additional psychological burden for the already stressed children and families [14]. In fact, since the majority of FN cases are diagnosed as fever of unknown origin, early initiatives in the United States as early as the 1990s aimed to reduce the length of hospitalization and to implement shorter antimicrobial treatment regimens in children with FN [10]. Efforts have been made to establish criteria for identifying low-risk FN episodes in which early discontinuation of EAT may be applied. However, these criteria vary substantially between different studies and institutions. Current guidelines for children with cancer and FN strongly recommend the cessation of EAT after 48 h of negative blood cultures (BCs) in children who remain afebrile for >24 h and have evidence of bone marrow recovery (BMR). However, cessation of EAT without evidence of BMR is considered as an option only for low-risk FN episodes after 48 h of negative BCs and 24 h of defervescence, with a conditional recommendation supported by moderate quality of evidence [15]. This narrative review aims to summarize the current evidence on the early discontinuation of EAT in children with cancer and FN, as well as the published data evaluating early discharge strategies with continuation of per os (PO) or intravenous (IV) antibiotics in the outpatient setting.

## 2. Materials and Methods

A comprehensive literature search for relevant articles was performed in Pubmed and Medline until 31 October 2025. The search strategy included the following terms: “early discontinuation”, “early discharge”, “short antibiotic courses”, “outpatient management”, “febrile neutropenia”, “cancer”, “pediatric cancer”, “children”, “pediatric”. The exact search strategy is presented in Appendix A.

Original prospective or retrospective cohort studies and randomized controlled trials (RCT) were included. Systematic reviews were excluded but the referenced studies were also screened for inclusion. Only articles in English were considered. Studies describing outpatient management strategies or FN treatment protocols were excluded if they did not specifically evaluate either EAT after initial intravenous treatment or EHD following a predefined short in-hospital treatment period. In particular, studies focusing on primary outpatient management from presentation, predefined standard-length antibiotic regimens, or general FN treatment approaches without a clearly defined early discontinuation or early discharge component were not considered eligible for inclusion. The primary outcome for evaluation was safety, estimated by readmission rates, morbidity and mortality associated with established treatment strategies. Secondary outcomes considered for appraisal were benefits for patients, such as reductions in LOS and duration of antibiotic therapy. These outcomes were extracted and qualitatively synthesized. An initial search was conducted and after deduplication, titles and abstracts were screened for relevance. After the initial literature search, the potentially eligible articles were retrieved and a full-text screening was performed to evaluate each article for inclusion. Furthermore, the reference lists of the included studies were screened to identify any additional relevant studies. Study selection was performed independently by two reviewers (S.P. (Smaragda Papachristidou) and G.P.). A senior expert (D.D. (Dimitra Dimopoulou)) verified the selection progress and resolved any potential disagreements. The final approval of the included studies was independently made by three reviewers (S.P. (Smaragda Papachristidou), D.D. (Dimitra Dimopoulou) and G.P.).

Although this review followed a structured and comprehensive literature search strategy and used a PRISMA flow diagram to transparently report study identification and selection, it was designed as a narrative review. No formal risk-of-bias assessment, meta-analysis, or quantitative synthesis was performed. The included studies were synthesized qualitatively, with an emphasis on clinical characteristics, safety outcomes, and practical implications, in order to provide a comprehensive narrative overview of the available evidence.

## 3. Results

### 3.1. Study Selection

**Figure 1:**
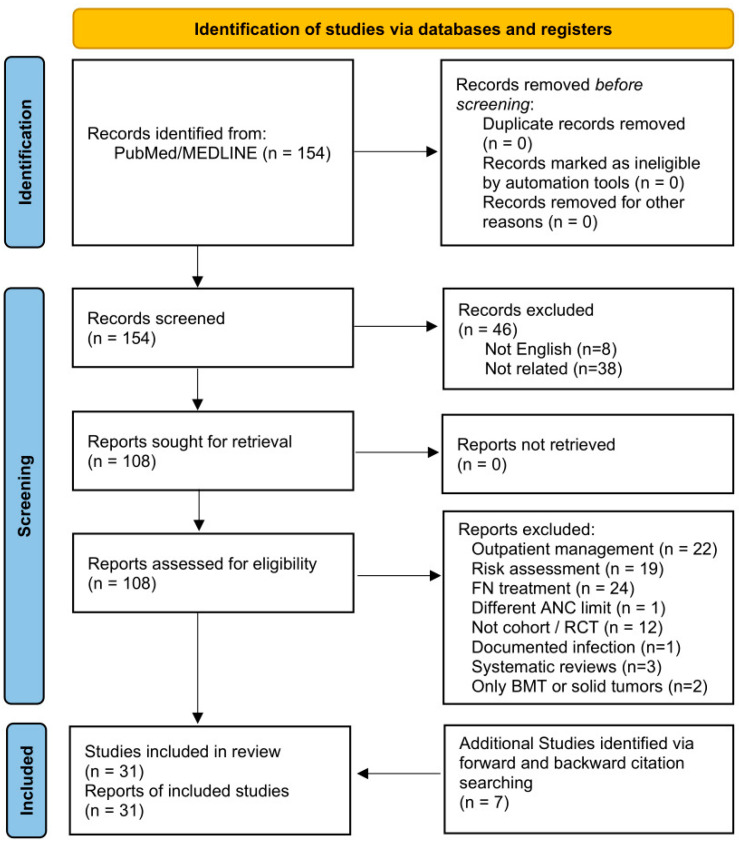
A PRISMA flow diagram of the included studies.

A total of 154 studies were identified after the initial literature search. Following the initial screening, 46 studies were excluded. Full-text evaluation was conducted for 108 studies. Among the excluded full-text articles, 22 studies labeled as “outpatient management” and 24 studies describing “FN treatment” were excluded because they did not meet the predefined criteria for early discontinuation of EAT or EHD, as they lacked a defined in-hospital treatment phase followed by early cessation of antibiotics or early discharge. Finally, 31 studies were included after evaluation for eligibility and relevance with the conducted research topic. The search is reported according to PRISMA guidelines [16]. The flow diagram is presented in Figure 1.

### 3.2. Study Characteristics

**Figure 2:**
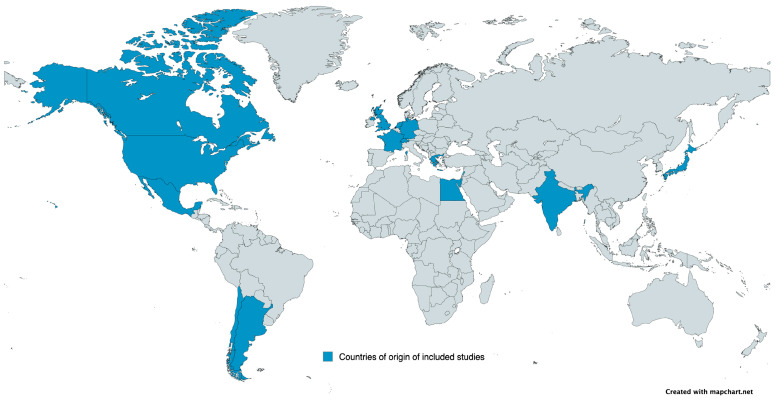
Countries of origin of included studies (map created with MapChart.net (https://www.mapchart.net/), access on 1 June 2025).

The publication characteristics of the selected studies are presented in Table 1 and their geographical distribution is shown in Figure 2. The included studies were published between 1990 and 2025 and originated from various regions worldwide, with the majority conducted in the United States (13 studies). Regarding the included studies’ design, thirteen studies were retrospective cohort studies, nine studies were prospective cohort studies, eight studies were RCT and one study had both retrospective and prospective arms. Twenty-one studies investigated the early discontinuation of EAT as a treatment strategy, whereas ten studies evaluated the early discharge with the continuation of PO or IV antibiotics in the outpatient setting. This narrative review focuses primarily on the safety of early EAT discontinuation and early discharge in pediatric oncology patients with FN. Secondarily, data on potential benefits from early discontinuation of EAT or early discharge for children with cancer and FN, such as reduced hospitalization duration, lower antimicrobial exposure, and healthcare cost, were extracted and synthesized for the current review. Detailed characteristics of all included studies are presented in Table 1, Table 2 and Table 3.

## 4. Discussion

### 4.1. Early Discontinuation of Antibiotics (EDA)

#### 4.1.1. Retrospective Studies About Safety

Since the 1990s, efforts have been made to shorten treatment regimens in children with cancer and FN. In 1990, a retrospective study from Dallas evaluated 114 episodes of FN. Of these, 77 episodes received early discontinuation of EAT after 24 h of defervescence with negative BCs for 48 h and evidence of BMR. Three readmissions (3.9%) due to recurrent fever were observed in children with early discontinuation of EAT compared with two readmissions (5.4%) among the 37 children who were hospitalized until neutropenia resolution [17].

Similarly, in 1992, a retrospective study from the United States reported early discontinuation of EAT in 70 out of 107 FN episodes (65%) observed in pediatric oncology patients. EAT was discontinued 24 h after defervescence, provided that the child remained clinically stable and BCs were negative for 48 h. All children, except for one case, had BMR trends. All had ANC < 0.5 × 10^3^/μL and 24 of them had ANC < 0.1 × 10^3^/μL. Notably, none of the 69 children with evidence of BMR experienced recurrence of fever [18].

Two retrospective studies from Dallas published in 1997 investigated the safety of early EAT discontinuation in children with low- and high-risk FN episodes. In the first study, low-risk episodes were defined as episodes of FN in children who appeared clinically well, remained afebrile for more than 24 h, had negative BCs and showed signs of BMR. In cases of localized infection, resolution of clinical signs of inflammation was also required. BMR was defined as an increase in platelet counts (PLT) and ANC. Among 580 FN episodes, 330 met the low-risk criteria, while the remaining episodes constituted the control group. PO antimicrobial therapy was continued in 81 cases (25%). There were 21 readmissions (6%), only 6 of which had evidence of BMR. Six children with EDA who were readmitted had bacteremia, a rate not statistically different compared to the control group [23]. In the second study by the same group, the same methodology was applied, but only children with prolonged neutropenia (>7 days) after EDA were evaluated. Among 33 cases out of 339 FN episodes, readmission was observed in two children (6%), who experienced worsening of localized infection. The readmission rate did not differ from the corresponding study of children with low-risk FN episodes, supporting the safety of EDA even in prolonged neutropenia when BMR is documented [22]. In 2002, a retrospective study from a center in Germany assessed EDA in children with cancer and FN without continuation of PO antibiotics after discharge. This change in institutional FN treatment protocol also compared the effectiveness of the combination therapy with ceftazidime and teicoplanin with imipenem monotherapy. Out of 106 episodes of FN, EDA was applied in 84 children, who did not develop a confirmed infection. Of those, 43 received a combination of ceftazidime and teicoplanin, while 41 children received imipenem monotherapy. No readmissions due to recurrence of fever were observed and no significant difference was found in the effectiveness of the two empirical antimicrobial treatment regimens [25]. In a retrospective study from Northern Alberta, Canada, published in 2005, children with FN and cancer, whose antimicrobial treatment was interrupted before BMR, were investigated. The decision for EDA was made by the treating clinical team. Out of the 217 FN episodes diagnosed as fever of unknown etiology, EAT was discontinued before neutrophil recovery in 112 of 199 patients (56%). Recurrence of fever was observed in only two patients, and no bacterial infections were subsequently identified [29]. A retrospective study from the United States published in 2016 explored the safety of EDA in children with FN who had received at least 48 h of antimicrobial treatment and remained afebrile for 24 h with negative BCs. During the study period, EDA was applied in 299 episodes of FN. Readmission due to recurrence of fever or infection was required in 50 cases (16.7%) with 27 new diagnoses of infection. Risk factors associated with readmission included ANC < 0.1 × 10^3^/μL (OR = 3.7 − 95%CI: 1.32–7.35) and initial diagnosis of acute lymphoid leukemia (ALL) (OR = 2.6 − 95%CI: 1.34–5.46). All patients who developed severe infection had ANC < 0.1 × 10^3^/μL at both the onset of FN and at the time of EAT discontinuation. Only one patient with severe infection required intensive care unit (ICU) admission, and no deaths were reported [33]. A retrospective study from the United States, published in 2017, investigated the threshold of ANC value considered safe for hospital discharge in children with cancer and FN. Among 350 episodes of FN, the risk of fever recurrence and bacteremia was calculated by ANC level at discharge. Readmission due to fever occurred in 3.9% (2 of 51 cases) of children with ANC between 0.1 × 10^3^/μL and 0.199 × 10^3^/μL at discharge, in 4.0% (5 of 125 cases) of children with ANC between 0.2 × 10^3^/μL and 0.499 × 10^3^/μL at discharge. In children with ANC above 0.5 × 10^3^/μL at discharge, readmission occurred in 5.0% (8 of 160 cases). In contrast, readmission was required in 14.3% (2 of 14 cases) of children with ANC less than 0.1 × 10^3^/μL at discharge. The only death in the series was recorded in a child with ANC = 0.29 × 10^3^/μL, who had achieved neutrophil count recovery and received a new chemotherapy regimen during hospitalization. The authors conclude that an ANC value > 0.1 × 10^3^/μL is a safe limit for the discontinuation of empirical antimicrobial therapy and discharge of children with FN [34]. In a retrospective study from Japan, published in 2020, risk factors for readmission after EDA in children and young adults with cancer and FN were assessed. Out of the 170 FN episodes in which EAT was discontinued early, 31 readmissions occurred due to recurrent fever, with 3 of them developing bacteremia. Multivariate analysis identified independent factors of increased risk for fever recurrence, such as incomplete remission of the initial disease, ANC < 0.011 × 10^3^/μL at the time of EDA, ANC > 0.061 × 10^3^/μL at the FN onset, and fever beginning within 1 day after the neutropenia onset [36]. In the same year, a retrospective study from the United Kingdom, evaluated randomly selected episodes of FN, in which EDA was implemented according to the center’s policy. EDA was applied in children with a good clinical appearance who remained afebrile for 48 h and had negative BCs. Among the 179 FN episodes analyzed, EDA was performed in 125 (70%). Readmission due to fever was recorded in seven cases (5.6%) and no death was reported [37]. In a retrospective study from the United States published in 2021, 131 episodes of EDA were analyzed, out of 729 total episodes of FN. EAT discontinuation occurred within the first 4 days after admission in children who remained afebrile with negative BCs. Eleven readmissions (8%) were recorded, two due to positive BCs and nine due to recurrence of fever. Mean ANC at the time of EDA was lower in children who required readmission (0.069 × 10^3^/μL) than in those who successfully completed EDA without complications (0.196 × 10^3^/μL). The patients who were readmitted were successfully treated and no severe outcomes were reported [38].

A retrospective study from the United States published in 2023 evaluated the safety of EDA in children with FN. At the single center, the policy for FN treatment was the discontinuation of EAT and discharge of the patient after 24 h of defervescence and 48 h of negative BCs. Out of the 1230 FN episodes recorded during the study period, EDA was performed in 765 (62%). Readmission within 7 days was observed in 122 children (15.9%). Identified risk factors for readmission included the diagnosis of acute myeloid leukemia (AML) and an ANC value < 0.1 x10^3^/μL at discharge. Among the children readmitted, only ten (1.31% of all children with EDA) had a positive BC and five (0.7%) were admitted to the ICU within 24 h of readmission [41].

In a retrospective study from France published in 2024, the safety of EDA in children with HR FN was evaluated, including all episodes of FN in children with prolonged neutropenia (>10 days), even those diagnosed with fever of unknown etiology. Out of the 51 episodes that met the eligibility criteria, EAT was discontinued early in 19 (37%). Recurrence of fever was recorded in 14 episodes (27%). No death or ICU admission was observed. Mucositis was a significant risk factor for fever recurrence [44].

#### 4.1.2. Prospective Studies About Safety

A prospective study from Texas published in 1994 evaluated children with FN who remained afebrile for more than 24 h, were clinically stable, demonstrated improvement of localized infection, if any, and had signs of BMR, despite persistent neutropenia. EDA was implemented in 82 episodes of FN (82/131 episodes of FN; 63.9%) and 78 of these children were discharged immediately. Among them, 51 cases had ANC < 0.2 × 10^3^/μL, 30 had ANC < 0.1 × 10^3^/μL and 6 had ANC = 0.0 × 10^3^/μL. Eight discharges represented protocol violations as patients did not show evidence of BMR, while readmission due to fever recurrence was reported in six of these cases (75%). Only one patient with confirmed BMR required readmission due to fever recurrence (1/70; 1.4%). Thirty children had localized infection and continued PO antibiotics at home [19]. In the same year, another study from the United States assessed the safety of EAT discontinuation after 48 h with negative BCs and 24 h of defervescence in children who continued to be neutropenic without clinical signs of infection. EAT was discontinued in 83 of the 231 FN episodes. Of these, 50 occurred in children with solid tumors, leukemia in remission, or other hematological disorders (group 1) and 33 of them in children with active leukemia (group 2). Recurrence of fever before neutrophil recovery was observed in 6% of cases in group 1 and 45% of cases in group 2, with five patients in group 2 showing active infection [20].

A prospective study from Denver published in 1995 investigated EDA 24 h after defervescence and negative BCs for 48 h, following an institutional update in the FN management protocol. This study included 32 episodes of FN with EDA. The mean ANC at admission was 0.06 × 10^3^/μL, increasing to 0.16 × 10^3^/μL at the time of antimicrobial therapy discontinuation. The mean duration of treatment was 3 days. Four children were readmitted to the hospital due to recurrence of fever and two children due to positive BCs while afebrile. Notably, none of the children who received EDA developed any serious complication [10].

In a multicenter, national-level study from the Netherlands in 2016, interleukin 8 (IL-8) levels were used to define risk groups and guide treatment decisions in children with FN. Children at high risk (HR) for bacterial infection continued EAT while IL-8 levels were assessed in the remaining children. Children with low IL-8 levels were considered low risk (LR) and were discharged without antimicrobial therapy at 12 h of hospitalization. Those with elevated IL-8 levels were considered intermediate risk (IR) and continued IV therapy. After 72 h of IV treatment, EAT was discontinued, if the children remained afebrile for more than 24 h, showed no signs of focal infection and had negative BCs. No treatment failure was observed among children with an IR FN episode undergoing EDA. In contrast, among children with a LR FN episode, six cases (12.8%) of treatment failure were observed, including recurrence of fever or occurrence of bacteremia due to coagulase (−) *staphylococcus* spp. [32].

In 2025, a study from Israel evaluated the safety and efficacy of EDA in children with FN, with negative BCs for 48 h, who remained afebrile for 24 h. EDA was implemented by alteration of the center’s FN treatment protocol, and only LR FN episodes were evaluated for EDA. The prospective arm of the study included children treated with the new FN treatment protocol, whereas children without EDA were reported retrospectively. Treatment failure with recurrence of fever occurred in 12/72 (16.7%) episodes in the EDA group, with 1 episode of bacteremia, compared to 10/108 (9.2%) episodes receiving EAT until BMR. This difference was not statistically significant [45].

Finally, a prospective single-center study from Greece applied EDA for children with LR FN by implementing it as the new institutional FN treatment policy. Inclusion criteria were first-line treatment for ALL or solid tumor, no signs of severe infection (hemodynamic instability, disseminated intravascular coagulation, hypotension, oliguria, respiratory distress, capillary refill time > 3 s or severe rigors), no signs of focal infection, and PLT > 30 × 10^3^/μL at the time of EAT discontinuation. Exclusion criteria included AML, BMT, ALL in Phase Ia (ALL IC -BFM 09), malignancy in relapse or signs of severe infection/sepsis. All children received EAT with piperacillin/tazobactam or cefepime combined with an aminoglycoside. Children that met inclusion criteria for LR FN had EAT discontinued 48 h after the last fever spike if BCs were negative, regardless of ANC, and were discharged from the hospital. Historical controls were used as the control group. Readmission rates for both EDA and control groups were reported as 0%. No severe adverse events were reported during the neutropenia phase or within 30 days following EDA in either group [46].

#### 4.1.3. RCTs About Safety

A randomized controlled study from India published in 2021 compared EDA with continued PO antimicrobial therapy in children with LR FN. LR criteria included solid tumor or hematological malignancy in remission, absence of focal infection, a child able to receive PO treatment and absence of pneumonia, sepsis, vomiting, neurological disorders, reduced level of consciousness or central venous catheter infection. Additional criteria included expected ANC recovery in less than 10 days, normal renal function, normal liver function and absence of hemodynamic instability. Exclusion criteria included patients with solid tumors and bone marrow infiltration, patients who have already participated in the study, patients under antimicrobial prophylaxis, HIV(+) patients, patients who underwent bone marrow transplant (BMT) or patients with cancer recurrence. Children received EAT with cefoperazone/sulbactam with or without the addition of amikacin on an outpatient basis. Children who remained afebrile for more than 24 h with negative BCs and persistent neutropenia were randomized. Out of the 75 children who participated in the study, 37 were randomized to the PO antibiotic group and 38 to the EDA group. In each of the two groups, two events of fever recurrence were recorded but no child required hospitalization. Treatment success rates were estimated at 94.6% in the PO antibiotic group and 94.7% in the EDA group, while the absolute risk difference was estimated at 0.1%. Therefore, non-inferiority of treatment discontinuation was observed compared to continued treatment [39].

A randomized single-center study from India in 2023 assessed the use of procalcitonin as an indicator of safe selection of children with FN who could be candidates for EDA. Children with LR FN who remained afebrile for more than 24 h, had negative BCs and negative procalcitonin were randomized after completing 72 h of EAT. Patients were assigned either to an EDA group or to a control group that received antibiotics for 7 days or until neutrophil recovery. A total of 46 FN episodes were randomized (23 episodes in each group). Treatment failure was observed in two children (8.7%) in the intervention group and in one child (4.3%) in the control group (*p* = 0.55). No deaths were reported [42].In conclusion, from a clinical perspective, although treatment failure was reported in the included studies, the number of ICU admissions among children readmitted after EDA was low and no were reported. These findings suggest that EDA has a safety profile that appears non-inferior to the established treatment approaches.

The aforementioned details of all included studies are summarized in Table 2.

#### 4.1.4. Benefits for Patients

Apart from the safety outcomes of EDA, several studies explored any potential benefits of this strategy for children with FN. Bash et al. reported that the mean duration of antibiotic therapy in children discharged with ANC < 0.5 × 10^3^/μL was 5.0 days compared with 5.9 days in those discharged with ANC > 0.5 × 10^3^/μL [19]. Similar results were reported regarding the mean length of hospital stay (LOS) with 5.1 and 6.3 days, respectively [19]. In children with prolonged neutropenia, the mean LOS was 11 days [22].

In 1997, Aquino et al. from Dallas reported shorter hospital stay among children managed with EDA, with an average length of stay at 5.4 days, which was statistically significantly different from the average length of stay in the control group (8.5 days) [23].

The biomarker-based risk stratification used by Miedema et al. in the Netherlands showed a significant reduction in LOS across risk groups [32]. The mean LOS was 3.6 days in the LR group, 4.7 days in the IR group managed with EDA, 11.2 days in the IR group with continued IV antimicrobial therapy, and 12 days in the HR group (*p* < 0.001). Furthermore, a significant reduction in antibiotic use was reported. Mean antibiotic duration was 0.6 days in the LR group, 3.5 days in the IR group receiving EDA, 9.2 days in the IR group with continued IV antimicrobial therapy, and 8.6 days in the HR group [32].

Srinivasan et el. reported a shorter median duration of antimicrobial therapy in children receiving EDA compared to the control group (3 days vs. 7 days; *p* < 0.001) [42].

Clément et al. demonstrated that the application of European Conference on Infections in Leukemia (ECIL) guidelines for EDA in children with FN resulted in a median duration of antibiotic use of 5 days, compared with 12 days in children managed without EDA [44].

Asleh et al. demonstrated a non-significant reduction in length of antibiotic use between EDA group and BMR groups, with median values of 3 and 4 days, respectively [45]. Similar results were reported for LOS, with median values of 4 and 5 days, respectively. However, the median value of ANC at discontinuation was lower in l the EDA group (0.18 × 10^3^/μL) than inand the BMR group (1.23 × 10^3^/μL) [45].

Finally, Papachristidou et al. reported a lower median value of ANC at discontinuation in the EDA group compared with the control group (0.16 × 10^3^/μL vs. 0.522 × 10^3^/μL) [46]. The duration of antibiotic use was reduced by 5 days (median value 2 days in the EDA group vs. 7 days in the control group). LOS was also reduced by 5 days (median value 2 days in the EDA group vs. 7 days in the control group) [46].

### 4.2. Early Hospital Discharge (EHD)

#### 4.2.1. Retrospective Studies About Safety

EHD has been proposed as a safer alternative to EDA, since patients receive antibiotics in the outpatient setting. A retrospective study from India, published in 2022, evaluated children with FN covering a period of 16 years. Children were initially treated on an outpatient basis, with continuation of IV ceftriaxone and amikacin in a single dose daily until completion of at least 7 days of antimicrobial treatment and 5 days of defervescence. Inclusion criteria included children under 18 years of age with FN after chemotherapy. Exclusion criteria included recent initiation of chemotherapy (newly diagnosed children), palliative chemotherapy, and children undergoing BMT. Hospital admission criteria were age under 1 year, AML, toxic clinical appearance, need for transfusion, and persistence of fever for 48–72 h. Out of the 952 FN episodes during the study period, 877 (92.2%) were treated as previously described. HR FN episodes were defined as those with neutropenia lasted for more than 7 days, episodes occurring after treatment for AML, malignancy not in remission, or poor clinical condition at the time of FN onset. Successful response to EAT with IV ceftriaxone and amikacin was recorded in 76.9% of FN episodes. The success rate was higher in low-risk (LR) FN episodes (85.7%) compared with HR FN episodes (65.5%; *p* < 0.0001). Following a response to initial antimicrobial therapy, fever recurred in 3/416 (0.7%) LR FN episodes and 43/253 (17%) HR FN episodes. Escalation of antimicrobial therapy was noted in 72 (15%) and 176 (4.5%) FN episodes in LR and HR groups, respectively (*p* < 0.0001) [40].

#### 4.2.2. Prospective Studies About Safety

A pilot study from Toronto published in 1994 investigated the discontinuation of IV antibiotics with transition to PO antimicrobial therapy in 23 children with FN. Eligible children were afebrile for more than 24 h, had negative BCs, ANC < 0.5 × 10^3^/μL, and no signs of sepsis. Out of 23 children, 3 readmissions were required, with initiation of IV antimicrobial therapy in 2 cases [21]. In 2002, a prospective single-center study from Seattle investigated early discontinuation of EAT and transition to PO therapy in children with a LR FN episode. All the children received initial EAT with IV ceftazidime. IV antibiotics were discontinued and PO treatment with ciprofloxacin plus amoxicillin was initiated, if the children were >4 years of age and weighed >16 kg, were able to comply with PO treatment, had already received IV EAT for 48 h, had negative BCs for more than 48 h, remained afebrile for more than 24 h, showed no signs of focal infection, and had no organ dysfunction. Out of the 350 FN episodes during the study period, 39 FN episodes met the criteria for EDA, and 30 were ultimately enrolled. Six children required readmission, including two who were unable to tolerate PO therapy and four who experienced treatment failure. All four treatment failures occurred in children with relapsed ALL. Three of these children developed recurrent fever and one experienced gastrointestinal bleeding related to chemotherapy. The three children with recurrent fever were diagnosed with hepatosplenic candidiasis, cellulitis and fever of unknown etiology, respectively, and were fully recovered upon readmission [26]. A prospective study from Mexico published in 2018 compared EDA with a historical control group. Children in the study group received PO ciprofloxacin, or in case of respiratory symptoms, they received PO amoxicillin/clavulanic acid, after 48 h of IV treatment as long as they were afebrile and did not exhibit PLT < 20,000/μL, Hb < 8 g/dL, active bleeding, or renal dysfunction. The study group included 37 patients who received EDA. Recurrence of fever was recorded in one EHD case (2.7%). Adverse events were reported in eight children with EHD (21.6%) compared to five children (12%) in the control group [35].

In a multicenter study from the United Kingdom published in 2023, the Australian–UK–Swiss Score (AUS) protocol was used, in order to assess the possibility of EHD with continued PO antibiotics. The AUS criteria are the following: (1) prior chemotherapy of greater intensity than ALL maintenance therapy (yes = 1, no = 0); (2) absolute white blood cell count < 0.3 × 10^3^/μL) (yes = 1, no = 0); and (3) platelet count < 50 × 10^3^/μL) (yes = 1, no = 0). The AUS value ranges from 0 up to 3 points. Using this approach, 46% of children were eligible for home administration of antibiotics and were discharged within 24 h compared to 2% of children with LR FN episodes who did not meet the AUS discharge criteria. Readmission occurred in 14% of children who were discharged, but no child was admitted to the ICU or died [43].

#### 4.2.3. RCTs About Safety

In 2001, a RCT from the United States compared IV EAT with PO cefixime in children with FN. The initial EAT was ticarcillin or ceftazidime in combination with vancomycin and tobramycin or amikacin. Exclusion criteria included allergy to cephalosporin or penicillin, colonization with *Pseudomonas aeruginosa* or *Staphylococcus aureus*, pneumonia, focal infection, hypotension, or severe mucositis. Children with FN were randomized once BCs were negative for 48 h. A total of 200 FN episodes were included; 100 were treated with IV antibiotics, while 100 were discharged with PO continued treatment with cefixime. A similar rate of treatment failure was observed, with 27 treatment failure episodes in the IV arm and 28 treatment failure episodes in the PO arm of the study [24]. In 2002, an RCT from a single center in Argentina compared continued IV EAT with PO antimicrobial therapy in children with LR FN. Children under 18 years of age with cancer and FN were included in the study if the family could support outpatient PO therapy. Exclusion criteria included bleeding, hypoglycemia, hypocalcemia, hypotension, impaired level of consciousness, renal dysfunction, hepatic dysfunction, respiratory failure, impaired general condition, cellulitis, severe mucositis, positive BC, expected neutropenia for more than 7 days, BMT, infection with ceftriaxone or ciprofloxacin-resistant microorganisms, allergy to ceftriaxone or ciprofloxacin, and inability of the family to adequately administer PO treatment at home. After the initial laboratory testing, the children were randomized into two groups. The first group received IV ceftriaxone and amikacin for 24 h followed by PO ciprofloxacin, while the second group received IV ceftriaxone and amikacin for 24 h and continued treatment with IV ceftriaxone as a monotherapy. All patients received antimicrobial treatment on an outpatient basis, which was discontinued when the ANC was above 0.1 × 10^3^/μL. Among the 557 FN episodes during the study period, 177 (32%) met the study inclusion criteria, 89 were randomized to the IV treatment group and 88 to the PO treatment group with comparable outcomes. Treatment failure was observed in four cases (5%) of the IV treatment group and in six cases (7%) of the PO treatment group. No deaths and no ICU admissions were observed [27]. In a multicenter RCT from Chile published in 2004, continuation of empirical IV antimicrobial therapy was compared with administration of PO therapy in children with FN and low risk of bacteremia. Exclusion criteria included bacteremia, positive culture from a sterile area, clinical or laboratory evidence of sepsis, and hemodynamic instability with organ dysfunction. All children received EAT with IV ceftriaxone and teicoplanin. Children who met the inclusion criteria were randomized into two groups: the group that continued IV antimicrobial therapy and the group that was discharged from the hospital with PO treatment with cefuroxime after 72 h of IV therapy, if the children remained afebrile. Out of the 390 FN episodes during the study period, 149 (38%) were randomized, with 71 to the IV treatment group and 78 to the PO treatment group. Treatment failure was observed in eight children (5% in the PO treatment group and 6% in the IV group). One death was observed in the IV treatment group, involving a child with neuroblastoma who developed bacteremia on the fourth day of hospitalization [28]. An RCT from Egypt published in 2007, including children with expected prolonged neutropenia (>7 days) and FN, compared continued IV therapy with ceftriaxone plus amikacin (C+A) on an outpatient basis, with continued IV therapy with imipenem in hospitalized children. Mortality rates were similar between the two groups, with an overall mortality rate of 3.4% [30]. In a multicenter RCT from Switzerland and Germany published in 2012, the safety and efficacy of continuing PO antimicrobial therapy in children with LR FN were evaluated. The study inclusion criteria were a diagnosis of cancer other than AML, mature beta-ALL and non-Hodgkin lymphoma, bone marrow involvement less than 25%, absence of signs of serious infection (hypotension, hypoxia, radiological picture of pneumonia, focal infection, positive BC, chills, temperature above 39.5 °C), ability to receive and continue PO treatment, creatinine value outside normal limits, and absence of known allergy to amoxicillin or ciprofloxacin. Out of the total 355 FN episodes, 62 were enrolled, with 34 children receiving continued IV antimicrobial treatment and 28 discontinuing IV antimicrobial treatment after 48 h of defervescence while receiving PO amoxicillin and ciprofloxacin at home. Oral treatment did not demonstrate inferiority to continued IV therapy regarding both safety (serious complication, death) and efficacy (no recurrence of infection, no treatment modification, absence of complications). No serious complications or deaths occurred in the PO group, whereas one death was reported in the IV group. Effective treatment outcomes were recorded in 23 of 27 children (85%) in the PO group and in 26 of 34 children (76%) in the IV group [31]. In conclusion, from a clinical perspective, two deaths were reported across two different studies, both occurring in the IV treatment arms. Furthermore, one study reported an equal mortality rate (3.4%) in both the hospitalized and discharged groups. Overall, these findings suggest that EHD has a safety profile that appears non-inferior to the established treatment approaches.

The findings from studies evaluating EHD strategies are summarized in Table 3.

#### 4.2.4. Benefits for Patients

Several studies have also assessed the potential benefits for children with FN undergoing EHD. Park et al. compared children who were successfully discharged early during the study with children who required readmission after being discharged early [26]. A statistically significant difference was observed in the duration of initial hospitalization (mean value 3.2 days versus 4.7 days, respectively). Significant differences were also noted in the ANC at discharge (mean value 0.071 × 10^3^/μL vs. 0.011 × 10^3^/μL respectively) and the duration of neutropenia (mean value 6.25 days vs. 16.3 days respectively) [26].

Paganini et al. reported a similar mean neutropenia duration between treatment groups, with 4.2 days in the IV treatment group and 4.7 days in the PO treatment group [27].

Ahmed et al. reported a mean LOS of 6.5 days in children treated with C+A on an outpatient basis, which was significantly lower than the mean LOS of 10.4 days in hospitalized children treated with imipenem [30]. A statistically significant difference was also observed in the duration of neutropenia, with a mean duration of neutropenia of 11.3 for the outpatient group and a mean duration of neutropenia of 12.3. for the hospitalized group [30].

In the study by Brack et al., the median LOS was 1 day for PO group compared with 4 days in the IV group [31].

Finally, Jackson et al., used the AUS criteria to assess EHD and reported an increase in mean LOS according to AUS [43]. Mean LOS values were 72, 96, 120 and 192 h for children with AUS values of 0, 1, 2 and 3, respectively [43].

In conclusion, although both EDA and EHD strategies were associated with a reduced length of hospital stay and antibiotic exposure, important differences between these approaches should be acknowledged. In EDA-only strategies, antibiotics are completely discontinued early, often resulting in shorter total antibiotic duration, whereas EHD strategies typically involve continuation of oral or intravenous antibiotics in the outpatient setting. Across the included studies, EDA was consistently associated with very short antibiotic courses (often 2–4 days) and minimal readmission rates in carefully selected low-risk patients, while EHD studies reported comparable safety outcomes but longer overall antibiotic exposure due to continued outpatient therapy. Direct quantitative comparisons are limited by heterogeneity and the absence of head-to-head trials.

### 4.3. Cost

Only a limited number of studies included in the current review have reported the estimated cost of FN episodes with EAT and/or the cost reduction from EAT or EHD in children with cancer and FN. In the prospective study from Texas published in 1994, the hospitalization cost analysis was available for 58 of 82 FN episodes. The average profit per patient was estimated at USD 5058 when EDA was applied [19]. Santolaya et al. calculated the difference in treatment costs between children who remained hospitalized and those who were discharged early [28]. The average treatment cost was USD 903 in the hospitalized group and USD 638 in the discharged group, representing a statistically significant difference (*p* = 0.003) [28]. Similary, Ahmed et al. reported a median hospitalization cost of USD 873 for children on an outpatient basis compared to USD 1655 for hospitalized children (*p* < 0.001) [30].

More recently, Papachristidou et al. reported a reduction in total hospital charges, insurance total cost, by EUR 286.9 (median value EUR 763.1 vs. EUR 1050.0 for EDA and control group, respectively) [46]. In a more detailed analysis, in the EDA group, the inpatient hospital stay cost, the antibiotic cost, the laboratory tests cost and the medical supplies cost was EUR 160, EUR 62.86, EUR 18.64, and EUR 8.72, respectively, resulting in a total estimated cost of EUR 309.76. On the contrary, in the control group, the inpatient hospital stay cost, the antibiotic cost, the laboratory tests cost, and the medical supplies cost was EUR 560, EUR 212.8, EUR 110.85, and EUR 27.02, respectively, resulting in a total estimated cost of EUR 924.69 [46].

## 5. Limitations

The narrative nature of this review, despite the use of systematic search methods, precludes formal quantitative comparison or pooled estimates of effect, and the findings should therefore be interpreted in the context of heterogeneity among study designs and patient populations. Another limitation emanates from the nature of the studied complication. Pediatric cancer remains rare, so the number of potential patients to be enrolled in studies is limited. Furthermore, RCTs may be difficult to be performed because of ethical and organizational constraints. These factors result in fewer patients included, different study designs and increased overall heterogeneity. Other factors contributing to the high heterogeneity among the included studies are the variability in the criteria used to define low-risk FN episodes, differences in the timing of EDA or EHD application and slight variations in neutrophil count thresholds. Collectively, the aforementioned limitations largely affect the strength of conclusions and the potential applicability to everyday clinical practice. In addition, from a clinical perspective, these factors limit the application of EDA or EHD, especially in healthcare systems with limited resources and in institutions, where free and direct access to readmission cannot be guaranteed.

## 6. Conclusions

EDA appears to be a safe strategy for FN treatment in children with FN and cancer, when certain criteria are met, including ≥24 h of defervescence and ≥48 h of negative BCs and absence of focal infection, toxic general appearance or hemodynamic instability. Although further studies are needed, EDA seems beneficial for children with FN and cancer by reducing both the LOS and the duration of antibiotic use. EHD with continuation of PO antibiotics also appears to be a safe strategy in selected low-risk patients, although it does not achieve the same degree of antibiotic exposure reduction as early discontinuation of EAT. However, the application of current strict low-risk FN criteria to ensure patient safety effectively excludes the majority of FN episodes from eligibility for EDA or EHD. This can be attributed to the potentially lethal nature of FN and to clinicians’ reluctance to adopt equivocal criteria. The process of adding more low-risk FN criteria is, therefore, conservative in order to avoid medical or ethical concerns. Future studies should provide higher-quality data, streamline and homogenize existing low-risk criteria, and use novel approaches, criteria, or biomarkers that may lead to broader patient selection, while consistently ensuring patient safety and efficiency in healthcare systems’ resource allocation and consumption.

## Figures and Tables

**Table 1:** Publication characteristics of included studies.

	Author	Type of Study	Outcome Studied	Study Period	Publication Date	Country
1	Mullen et al. [17]	Retrospective	EDA	02/1988–02/1989	1990	USA (Dallas)
2	Griffin et al. [18]	Retrospective	EDA	04/1989–11/1989	1992	USA
3	Bash et al. [19]	Prospective	EDA	11/1989–07/1990	1994	USA (Texas)
4	Jones et al. [20]	Prospective	EDA	01/01/1987–01/09/1992	1994	USA
5	Lau et al. [21]	Prospective	EHD	10/1990–07/1991	1994	Canada (Toronto)
6	Cohen et al. [10]	Prospective	EDA	01/09/1990–01/05/1991	1995	USA (Denver)
7	Aquino et al. [22]	Retrospective	EDA	11/07/1991–04/02/1994	1997	USA (Dallas)
8	Aquino et al. [23]	Retrospective	EDA	06/1992–05/1995	1997	USA (Dallas)
9	Shenep et al. [24]	Randomized Controlled Trial (RCT)	EHD	24/01/1991–02/06/1995	2001	USA
10	Lehrnbecher et al. [25]	Retrospective	EDA	01/1994–06/1996	2002	Germany
11	Park et al. [26]	Prospective	EHD	05/1998–05/1999	2003	USA (Seattle)
12	Paganini et al. [27]	RCT	EHD	08/2000–04/2002	2003	Argentina
13	Santolaya et al. [28]	RCT	EHD	01/06/2000–28/02/2003	2004	Chile
14	Hodgson-Viden et al. [29]	Retrospective	EAD	01/06/1997–01/07/2002	2005	Canada
15	Ahmed et al. [30]	RCT	EHD	9-month period	2007	Egypt
16	Brack et al. [31]	RCT	EHD	01/2004–12/2007	2012	Germany/Switzerland
17	Miedema et al. [32]	Prospective	EDA	06/2006–01/2012	2016	Netherlands
18	Villanueva et al. [33]	Retrospective	EDA	01/01/2006–31/01/2012	2016	USA
19	Campbell et al. [34]	Retrospective	EDA	2007–2012	2017	USA
20	Gil-Veloz et al. [35]	Prospective	EHD	01/2012–12/2013	2018	Mexico
21	Kobayashi et al. [36]	Retrospective	EDA	04/2012–03/2016	2020	Japan
22	Seneviratne et al. [37]	Retrospective	EDA	2009–2012	2020	UK
23	Huschart et al. [38]	Retrospective	EDA	09/2005–10/2016	2021	USA
24	Kumar et al. [39]	RCT	EDA	01/2017–12/2018	2021	India
25	Kanvidne et al. [40]	Retrospective	EHD	01/2002–12/2017	2022	India
26	Peters et al. [41]	Retrospective	EDA	2014–2019	2023	USA
27	Srinivasan et al. [42]	RCT	EDA	02/2020–10/2021	2023	India
28	Jackson et al. [43]	Prospective	EHD	17/04/2020–19/04/2021	2023	UK
29	Clément et al. [44]	Retrospective	EDA	01/01/2017–31/12/2021	2024	France
30	Asleh et al. [45]	Retrospective/ Prospective	EDA	01/01/2017–30/11/2022	2025	Israel
31	Papachristidou et al. [46]	Prospective	EDA	01/02/2017–30/11/2020	2025	Greece

EDA: Early Discontinuation of Antibiotics; EHD: Early Hospital Discharge; RCT: Randomized Controlled Trial; UK: United Kingdom; USA: United States of America.

**Table 2:** The characteristics of studies evaluating the clinical impact of early antibiotic discontinuation in pediatric oncology patients with febrile neutropenia.

	Author	Control Group	FN Episodes	EDA Patients	Afebrile (Hours)	IV Antibiotic Duration (Hours)	Treatment Failure/ Readmission ^#^	Mean ANC at EDA (×10^3^/μL)	Mean Antibiotic Duration (Days)	Mean Hospitalization Duration (Days)
1	Mullen et al. [17]	BMR group vs. EDA	114	77 (67.5%)	24	ND *	S ^†^: 3/77 (3.9%) C ^‡^: 2/37 (5.4%)	ND	ND	ND
2	Griffin et al. [18]	No	107	70 (65%)	24	ND	No patients with BMR	ND	ND	ND
3	Bash et al. [19]	ANC > 0.5 × 10^3^/μL group vs. EDA	131	82 (63.9%) 78 hospital discharge	24	ND	6/8 protocol breach-no BMR 1/70 (1.4%)	0.16	S: 5.0 C: 5.9	S: 5.1 C:6.3
4	Jones et al. [20]	Group 1 vs. Group 2	231	83 (50 solid tumors and leukemia in remission Group 1–33 active leukemia Group 2)	24	ND	Group 1—6% Group 2—45% 5—active infection	ND	ND	ND
5	Cohen et al. [10]	No	ND	32	24	48	4/32—fever 2/32—BC (+) without fever No serious complication	ND	3 days	ND
6	Aquino et al. [22]	EDA group vs. children who remain hospitalized	339	33 (9.7%)	ND	ND	2/33 (6%) for worsening focal infection	ND	ND	11
7	Aquino et al. [23]	EDA group vs. children who remain hospitalized	580	330 (56.9%)	ND	ND	21/330 (6%) 6 with BC (+) (same as controls)	0.156 × 10^3^/μL	ND	S: 5.4 C:8.5
8	Lehrnbecher et al. [25]	No	106	84 (79.2%)	24	72	0/84% (0%)	ND	ND	ND
9	Hodgson-Viden [29]	No	217	112 (51.6%)	ND	ND	2/112 (1.8%) No bacterial infection	0.4^§^	ND	ND
10	Miedema et al. [32]	IL-8 use LR vs. MR vs. HR	ND	233 LR-47 MR-122 HR-64	(MR) 24	LR—no antibiotic MR-72	LR—6/47 (12.8%)— 1 BC (+) MR—0/122 (0%)	ND	LR: 0.6 MR: 3.5 MR-IV: 9.2 HR: 8.6	LR: 3.6 MR: 4.7 HR: 12
11	Villanueva et al. [33]	No	ND	299	24	48	50/299 (16.7%) 27 new infections 1 ICU No death	ND	ND	ND
12	Campbell et al. [34]	Safe limit of discontinuation	350	ND	24	ND	<100: 2/14 (14.3%) 100–199: 2/51 (3.9%) 200–499: 5/125 (4%) >500: 8/160 (5%)	ND	ND	ND
13	Kobayashi et al. [36]	Recurrent vs. no recurrent fever	434	170 (39.1%)	ND	ND	31/170 fever 3/170 BC (+)	Recurrence: 0.048 No recurrence: 0.095	Recurrence: 13 No recurrence: 13	ND
14	Seneviratne et al. [37]	No	179	125 (70%)	48	48	7/125 (5.6%) no death	ND	ND	ND
15	Huschart et al. [38]	Readmission vs. no readmission	729	131 (18%)	ND	<96	11/131 (8%) 2 BC (+) No death	Readmission: 0.069 No readmission: 0.196	ND	Readmission: 3.09 No readmission: 3.29
16	Kumar et al. [39]	EDA vs. PO	255 patients	75 Randomized 37 PO 38 EDA	24	ND	2/37 PO 2/38 EDA Fever without readmission	ND	ND	ND
17	Peters et al. [41]	Readmission vs. no readmission	1230	765 (62%)	24	ND	7/122 (15.9%) 10 BC (+) 5 ICU	ND	ND	ND
18	Srinivasan et al. [42]	EDA vs. continuation of antibiotics/procalcitonin	ND	46 Randomized 23 EDA, 23 IV	24	72	EDA: 2/23 (8.7%) Antibiotic: 1/23 (4.3%) no death	ND	EDA: 3 Antibiotic: 7	ND
19	Clément et al. [44]	Followed or not ECIL guidelines ^¶^	ND	19	24–48	72	14 (27%) no ICU no death	ND	ECIL ^§^: 5 No ECIL ^§^: 12	ND
20	Asleh et al. [45]	EDA vs. BMR	367	72	24	ND	EDA: 12/72 (16.7%) BMR: 10/108 (9.2%)	EDA: 0.18 BMR: 1.23	EDA: 3 BMR: 4	EDA: 4 BMR: 5
21	Papachristidou et al. [46]	EDA vs. historical controls	456	36	24	48	0/36 (0%)	EDA: 0.16 C: 0.522	EDA: 2.0 C: 7.0	EDA: 2.0 C: 7.0

* ND denotes No Data. ^†^ S denotes the study group. ^‡^ C denotes the control group. ^§^ denotes that the value is a median and not a mean. ^¶^ ECIL guidelines: published and endorsed by the European Conference on Infections in Leukemia. ^#^ Composite outcome including one or more of the following events, as reported in the individual studies: recurrence of fever after initial response, documented bloodstream infection or other microbiologically confirmed infection, need for escalation or modification of antimicrobial therapy, unplanned hospital readmission, or admission to the intensive care unit, death. ANC: Absolute Neutrophil Count, BC: Blood Culture, BMR: Bone Marrow Recovery, ECIL: European Conference on Infections in Leukemia, EDA: Early Discontinuation of Antibiotics, FN: Febrile neutropenia, HR: high risk, ICU: Intensive Care Unit, IV: Intravenous, LR: low risk, MR: medium risk, ND: No data, PO: Per Os.

**Table 3:** The characteristics of studies evaluating the clinical impact of early hospital discharge in pediatric oncology patients with febrile neutropenia.

	Author	FN Episodes	Study Population	Afebrile (Hours)	IV Duration (Hours)	Treatment Failure/ Readmission ^#^	Control Group	Mean Hospitalization Duration (Days)	ANC at Discharge (×10^3^/μL)	Mean Duration of Neutropenia (Days)
1	Lau et al. [21]	157	23	24	72	PO: 3/23 IV: 2/23	PO vs. IV	ND *	ND	ND
2	Shenep et al. [24]	433	100 IV 100 PO	ND	48–72	PO: 28/100 IV: 27/100	PO vs. IV	ND	ND	ND
3	Park et al. [26]	350	30	24	48	6/30 2 inability PO 3 fever 1 gastric hemorrhage	Success vs. Failure ^±^	Success: 3.2 Failure: 4.7	Success: 0.071 Failure: 0.011	Success: 6.25 Failure: 16.3
4	Paganini et al. [27]	557	177 R ^†^ 89 IV/88 PO	ND	24	PO: 6/88 (7%) IV: 4/89 (5%)	PO vs. IV	ND	ND	PO: 4.7 IV: 4.2
5	Santolaya et al. [28]	390	149 R 71 IV/78 PO	ND	24–36	PO: 4/78 (5%) IV: 4/71 (6%) 1 death IV with BC (+)	PO vs. IV	ND	ND	ND
6	Ahmed et al. [30]	262	129 R 66 C+A ^‡^ /63 IMI	(Defervescence) 72 afebrile:24	72	Mortality 3.4% both groups	Hospitalized vs. Discharged	C+A: 6.5 IMI: 10.4	ND	C+A: 11.3 IMI: 12.3
7	Brack et al. [31]	355	62 R 34 IV/28 PO	ND	24 h	PO: 4/27 (14.8%) IV: 8/34 (23.5%) 1 death IV group	PO vs. IV	PO ^§^: 1 IV ^§^: 4	ND	ND
8	Gil-Veloz et al. [35]	ND	37 PO 43 IV	ND	48	EHD: 1/37 (2.7%) Side effects EHD: 8/37 IV: 5/43	historical controls	ND	ND	ND
9	Kanvidne et al. [40]	952	877 (92.2%)	120	168	Fever LR: 3/416 (0.7%) HR: 43/253 (17%) Upgrade Antibiotics LR: 72 (15%) HR: 176 (4.5%)	LR vs. HR	ND	ND	ND
10	Jackson et al. [43]	729	ND	ND	AUS = 0: 4–8 AUS = 1: 4–24 AUS = 3: 24 AUS = 4: 48	14% readmission No ICU or death	AUS evaluation	AUS = 0: 3 AUS = 1: 4 AUS = 3: 5 AUS = 4: 8	ND	ND

* ND denotes No Data. ^†^ R denotes the total number of patients that were randomized in study groups. ^‡^ C+A denotes the combined administration of ceftriaxone plus amikacin. ^§^ denotes that the value is a median and not a mean. ^±^ Success: completion of early hospital discharge without recurrence of fever, need for antibiotic modification, readmission, or intensive care unit admission; Failure: occurrence of any of the above events requiring readmission or escalation of care. ^#^ Composite outcome including one or more of the following events, as reported in the individual studies: recurrence of fever after initial response, documented bloodstream infection or other microbiologically confirmed infection, need for escalation or modification of antimicrobial therapy, unplanned hospital readmission, or admission to the intensive care unit, death. AUS: Australian–UK–Swiss Score, C+A: ceftriaxone + amikacin, EHD: Early Hospital Discharge, HR: high risk, ICU: Intensive Care Unit, IMI: imipenem, IV: intravenous, LR: low risk, ND: No data, PO: Per Os.

## Data Availability

The original contributions presented in this study are included in the article. Further inquiries can be directed to the corresponding author.

## Abbreviations

The following abbreviations are used in this manuscript:

ALL	Acute Lymphoblastic Leukemia
AML	Acute Myeloid Leukemia
ANC	Absolute Neutrophil Count
AUS	Australian–UK–Swiss Score
BCs	Blood Cultures
BMR	Bone Marrow Recovery
BMT	Bone Marrow Transplant
C+A	Ceftriaxone + Amikacin
EAT	Empiric Antibiotic Therapy
ECIL	European Conference on Infections in Leukemia
EDA	Early Discontinuation of Antibiotics
EHD	Early Hospital Discharge
FN	Febrile Neutropenia
HR	High-Risk
ICU	Intensive Care Unit
IMI	Imipenem
IR	Intermediate Risk
IV	Intravenous
LOS	Length Of Hospital Stay
LR	Low-Risk
MR	Medium-Risk
ND	No Data
PLT	Platelet Count
PO	Per Os
RCT	Randomized Controlled Trial

## Appendix A

((early discontinuation) OR (early discharge) OR (short antibiotic courses) OR (outpatient management)) AND ((febrile neutropenia) OR ((fever) AND (neutropenia))) AND ((cancer) OR (pediatric cancer)) AND ((children) OR (pediatric))
